# Effectiveness and safety of anti-CGRP monoclonal antibodies in hemiplegic migraine: an individual patient quantitative analysis

**DOI:** 10.1186/s10194-026-02283-5

**Published:** 2026-01-30

**Authors:** Marina Romozzi, Matteo Palermo, Daisuke Danno, Masahito Katsuki, Federico Tosto, Francesco Signorelli, Catello Vollono, Yasuhiko Matsumori, Paolo Calabresi, Raffaele Ornello, Luigi Francesco Iannone

**Affiliations:** 1 Department of Neuroscience, Università Cattolica del Sacro Cuore, Rome, Italy; 2 Neurology Unit, Dipartimento di Neuroscienze, Organi di Senso e Torace, Fondazione Policlinico Universitario Agostino Gemelli IRCCS, Rome, Italy; 3 Headache Center, Department of Neurology, Tominaga Hospital, Osaka, Japan; 4 Insight Science Foundation Ireland Research Centre for Data Analytics, School of Human and Health Performance, Dublin City University, Dublin, Ireland; 5 Physical Education and Health Center, Nagaoka University of Technology, Niigata, Japan; 6 Department of Neuroscience, “Giovanni Paolo II” Hospital, Catanzaro, Italy; 7 Department of Neurosurgery, Fondazione Policlinico Universitario Agostino Gemelli IRCCS, Università Cattolica del Sacro Cuore, Rome, Italy; 8 Sendai Headache and Neurology Clinic, Sendai, Japan; 9 Department of Biotechnological and Applied Clinical Sciences, University of L’Aquila, L’Aquila, Italy; 10 Department of Biomedical, Metabolic and Neural Sciences, University of Modena and Reggio Emilia, Modena, Italy

**Keywords:** Familial hemiplegic migraine (FHM), ATP1A2, CACN1A, SCN1A, Galcanezumab, Fremanezumab, Erenumab

## Abstract

**Background:**

Hemiplegic migraine (HM) is a rare and disabling subtype of migraine with aura characterized by reversible motor weakness. Preventive treatment remains challenging, as patients with HM are systematically excluded from RCTs. Monoclonal antibodies targeting the calcitonin gene-related peptide pathway (anti-CGRP mAbs) have shown effectiveness in migraine with typical aura, but evidence in HM is limited.

**Methods:**

This systematic review and quantitative synthesis followed PRISMA guidelines. PubMed, Scopus, and Web of Science were searched for studies reporting patients with HM treated with anti-CGRP mAbs. Individual patient data were extracted, and pooled analyses were conducted for changes in monthly headache days (MHDs), days with aura, and Migraine Disability Assessment (MIDAS) score from baseline to three months. Risk of bias was assessed using the GRADE approach.

**Results:**

Six studies (four case series and two case reports), including 13 patients, met the inclusion criteria. MHDs decreased from 18 (IQR 15–25) at baseline to 9 (IQR 3–20) at three months; however, the numerical decrease was not statistically significant (*p* = 0.077). Median monthly days with aura decreased from 3 (IQR 2–8) to 0.6 (IQR 0–2.3; *p* = 0.018), and the MIDAS score decreased from 88 (IQR 66–154) to 28 (IQR 5–74; *p* = 0.018). Ten patients (83%) achieved > 50% reduction in MHDs and aura frequency, and nine (75%) > 50% MIDAS reduction. No adverse events were reported.

**Conclusions:**

Although the overall evidence certainty is very low, anti-CGRP mAbs appear to reduce aura burden and migraine-related disability in HM with good tolerability, supporting the need for prospective multicenter studies and registries to confirm their effectiveness and safety in this rare, highly disabling condition.

**Supplementary Information:**

The online version contains supplementary material available at 10.1186/s10194-026-02283-5.

## Introduction

Hemiplegic migraine (HM) is a rare form of migraine with aura that manifests with reversible motor weakness, along with reversible sensory, visual, or speech changes that can occur as a sporadic (SHM) or familial disorder [1]. Familial hemiplegic migraine (FHM) is an autosomal dominant disease with variable expressivity and reduced penetrance [2]. The International Classification of Headache Disorders-3 (ICHD-3) classifies FHM into three subtypes, based on the gene involved. A diagnosis of FHM is established when first- or second-degree relatives are affected by HM. Mutations in the *CACNA1* gene cause FHM1, encoding the α1A subunit of calcium channels [1]. Mutations in the *ATP1A2* gene, which encodes a catalytic subunit of a sodium/potassium ATPase, cause FHM2. FHM3 is caused by mutations in a sodium channel α-subunit coding gene, SCN1A [1].

Newly, a fourth gene, *PRRT2*, has been associated with FHM (classified as FHM4), which encodes a proline-rich transmembrane protein [3].

However, the established mutations likely account for a small percentage of cases of FHM, and other not yet identified genes are potentially involved [3].

The clinical presentation of the various forms of FHM is highly variable. Usually, HM is characterized by unilateral motor weakness occurring as an aura symptom, but additional symptoms may include visual field defects, scotoma, hemianopia, paresthesia, numbness, or ataxia. Motor symptoms typically begin in the hand and gradually spread to the arm and face, usually developing over 20–30 min. In rare cases, they can appear abruptly and mimic a stroke. Duration usually ranges from several hours to a few days, and complete resolution is the rule in most patients; however, in some patients, the weakness can be persistent. Headache is commonly associated, often overlapping with the aura phase, but sometimes beginning afterward. Severe episodes can be complicated by seizures, coma, encephalopathy, fever, cerebellar involvement, cerebral edema, or cerebral infarction [4–7].

In HM, headache is generally severe, and not only the headache but also the aura symptoms, including motor weakness, often result in substantial disability [7].

The pathophysiological mechanism underlying the motor weakness and other types of aura is likely the cortical spreading depression (CSD). Mutations related to HM increase neuronal excitability, leading to a higher susceptibility to CSD [2]. The role of CGRP in migraine aura is still a matter of debate and was recently reviewed in [8].

However, the monoclonal antibodies targeting CGRP or its receptor (anti-CGRP mAbs) are increasingly used in patients with migraine with typical aura, with consistent evidence of efficacy and effectiveness emerging from post-hoc analyses of randomized controlled trials (RCTs) as well as real-world studies [9–12]. These findings raise the hypothesis that a similar therapeutic effect might also be observed in patients with HM.

Therefore, we aimed to systematically review the available evidence on the effectiveness and safety of anti-CGRP mAbs in patients with HM and to conduct a quantitative synthesis on treatment outcomes.

## Methods

### Search strategy

This systematic review was performed according to Preferred Reporting Items for Systematic Reviews and Meta-Analyses (PRISMA) 2020 guidelines [13] to evaluate the effectiveness of anti-CGRP mAbs in patients with HM. A comprehensive literature search of PubMed, Web of Science, and Scopus was performed for studies published in English until September 8th 2025 using the following string: “(erenumab OR fremanezumab OR galcanezumab OR eptinezumab OR “AMG 334” OR “TEV-48125” OR “LY2951742” OR “ALD403” OR ((CGRP OR “calcitonin gene related peptide” OR “calcitonin gene-related peptide”) AND (antibod* OR “monoclonal” OR mAb* OR “receptor antibod*”))) AND (hemipleg* OR “hemiplegic migraine” OR “familial hemiplegic” OR FHM OR SHM OR (migraine AND (“motor aura” OR hemipares* OR weakness OR paresis)) OR (“migraine with aura” AND (motor OR weakness)) OR CACNA1A OR ATP1A2 OR SCN1A OR PRRT2 OR SLC4A4)”.

The research question was structured using a modified PICO framework, omitting the comparator as none was available. The Population was defined as individuals with HM, the Intervention as treatment with anti-CGRP mAbs, and the Outcomes as improvement in migraine clinical features and patient-reported scores.

### Eligibility criteria

The literature search included peer-reviewed studies published in English, with no date limitations. Randomized clinical trials, observational studies, case series, and case reports were considered for inclusion. Only articles reporting quantitative data were included.

Eligible studies described patients diagnosed with HM in accordance with the ICHD-3 criteria and treated with anti-CGRP mAbs. We excluded studies lacking confirmed diagnoses, failing to specify patient numbers, discussing other forms of migraine, or not providing clear evidence of anti-CGRP mAb treatment. Review articles and papers without relevant clinical data were also excluded. Both familial and sporadic forms of HM were considered eligible.

### Selection process

After searching the three databases, all results were collected. Duplicates were removed using the Rayyan software [14]. Two authors (M.P. and F.T.) independently screened titles and abstracts. Additional publications were retrieved from the reference list of relevant articles. Three reviewers (F.T., M.P., and F.S.) independently screened the full texts of the articles that were selected, with a strong inter-rater agreement (Cohen’s kappa 0.81); a senior author (M.R.) resolved discrepancies.

**Fig. 1:**
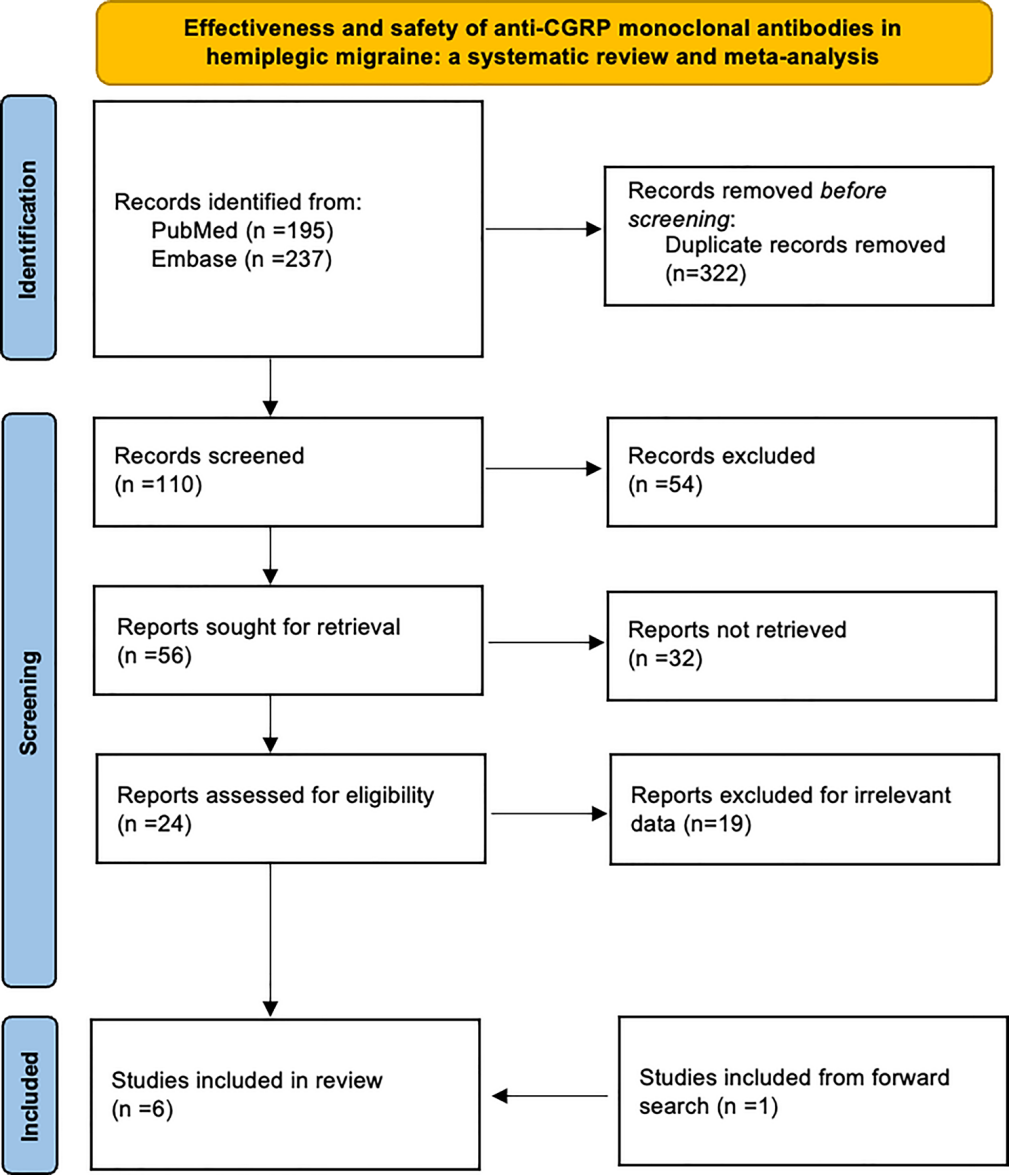
PRISMA flowchart

A PRISMA flowchart depicting the selection and screening process is provided in Fig. 1.

### Data extraction

Two Reviewers (F.T. and M.R.) independently extracted all data using a piloted data collection form, and discrepancies were resolved through consensus.

We recorded the first author and year of publication of each included article, as reported in Table 1. Subsequently, we identified the mutated gene and the specific clinical features of HM described for each patient. We then extracted treatment-related information, including the type of anti-CGRP mAb administered and the duration of therapy, outcome data, and adverse events. We adopted an individual patient data approach in which data from each single patient were collected and pooled together.

Regarding migraine outcomes, we recorded the change in monthly headache days (MHDs), monthly days with aura, and Migraine Disability Assessment (MIDAS) score from baseline to the third month of treatment, the proportion of patients with a > 50% reduction in MHDs from baseline to the third month of treatment, the proportion of patients with a > 50% reduction in monthly days with aura from baseline to the third month of treatment, and the proportion of patients with a > 50% reduction in MIDAS score from baseline to the third month of treatment.

**Table 1:** Clinical studies on effectiveness and safety of anti-CGRP monoclonal antibodies (mAbs) in patients with hemiplegic migraine

First Author (Year)	Study design (number of patients treated)	Mutation	Type of HM	Type of anti-CGRP mAb	Treatment duration (months)	Outcome	Adverse events
Danno (2023)	Case series (6)	Not reported	4 SHM, 2 FHM	Galcanezumab	3	3/6 had ≥ 50% reduction in moderate–severe headache days, 4/6 had ≥ 50% reduction in motor aura days, 5/6 improved at PGIC/MIDAS	None
D’Apolito (2024)	Case report (1)	*SCN1A p.Ser628Thr*	SHM	Galcanezumab	12	Complete disappearance of hemiplegic aura, MIDAS dropped from 168 to 4, marked benefit from 3 months onward	None
Antenucci (2025)	Case report (1)	*CACNA1A*	SHM	Eptinezumab	6	Reduction of 58% monthly headache days, reduction of 67% motor aura attacks, MIDAS dropped from 152 to 68, improved VAS	None
Indelicato (2024)	Case report (1)	*CACNA1A T666M*	FHM1	Galcanezumab	6	Headache days reduced from 4/month to 1/month	None
Sottani (2025)	Familial case series (3)	*PRRT2 c.649dupC*	FHM	Galcanezumab	6	Headache days dropped from 11.7 to 1.7/month, all had ≥ 50% reduction in aura frequency; no specified reduction of MIDAS and acute meds	None
Héja and Oláh (2025)	Case report (1)	Not tested	HM (not specified)	Fremanezumab	11	At 12 weeks: 3–4 headache days with milder severity; at 11 months: 3.6 headache days, MIDAS dropped from 168 to 5	None

CGRP, calcitonin gene-related peptide; FHM, familial hemiplegic migraine; HM, hemiplegic migraine; PGIC, Patients’ Global Impression of Change; SHM, sporadic hemiplegic migraine; n, number; MIDAS, Migraine Disability Assessment; VAS, visual analogue scale

### Quality assessment

We assessed the certainty of the evidence using the GRADE (Grading of Recommendations, Assessment, Development and Evaluation) approach. Risk of bias for case reports and case series was assessed using the Joanna Briggs Institute (JBI) Critical Appraisal Checklists. We evaluated the domains of patient selection, clarity of clinical history and timeline, diagnostic methods, intervention details, and outcome reporting. Based on these criteria, each study was categorized as having a serious or critical risk of bias. This judgement was subsequently incorporated into the GRADE evidence profiles.

Any disagreements were solved through discussion and consensus.

### Statistical analysis

Outcome measures varied across the included case reports and case series. For this reason, we adopted an individual patient data approach, extracting and pooling only those outcomes that were consistently reported across multiple studies.

We pooled continuous outcomes as medians and interquartile ranges, while we reported binary outcomes as numbers and proportions together with 95% confidence intervals calculated according to Poisson distributions. Comparisons between baseline and three months were performed via the Wilcoxon test. All the outcomes not included in pooled analyses were reported descriptively in the text. Analyses were performed using R, version 4.2.2, and RStudio, version 2025.05.1 + 513.

## Results

### Study selection

The literature search retrieved a total of 432 records from PubMed, Scopus, and Web of Science. After removal of duplicates (*n* = 322), 110 unique articles were screened. Of these, 54 were excluded based on title and abstract review, while 56 were retained for full-text assessment. Following a detailed eligibility evaluation, six studies met the inclusion criteria and were incorporated into the systematic review on the use of anti-CGRP mAbs in patients with HM (Fig. 1).

### Studies characteristics

The final set of included studies (*n* = 6) consisted of four case reports, each describing a single patient, and three case series including two, three, and six patients, respectively, for a total of 13 patients treated with anti-CGRP mAbs. All the studies’ features are summarized in Table 1. Patients included were recruited from tertiary-care neurological settings, predominantly specialized headache clinics or university-affiliated neurology departments. The studies originated mainly from Europe (Italy and Hungary) and Japan, and all involved centers with specific expertise in migraine and rare headache disorders.

Baseline frequencies of hemiplegic (motor) aura and non-motor aura were not systematically reported across the included studies and were therefore described under the broader categories of MHDs or days with aura when available. Episodes of migraine without aura or with typical (non-motor) aura were reported in some patients; however, their baseline and post-treatment frequencies were inconsistently documented and were therefore included within overall MHDs when available.

Antenucci et al. reported a case of sporadic HM with the very uncommon deletion in *CACNA1A* located on exon 47. The patient, previously a non-responder to multiple preventive therapies, experienced a meaningful improvement after eptinezumab infusion. The study showed a 58% reduction in headache days and a 67% reduction in motor aura attacks at six months, with improved Visual Analogue Scale (VAS) (from 9 to 5), MIDAS score (from 152 to 68), decreased NSAID use (from 19 to 6 days/month), and a “*very much improved*” Patient Global Impression of Change (PGIC) score at follow-up. No adverse events were reported [15].

D’Apolito et al. described a case of sporadic HM associated with a novel missense mutation in the *SCN1A* gene (*p.Ser628Thr*). This patient presented with ≥ 8 attacks/month (not specified if MHDs or monthly migraine days), all preceded by aura (approximately one per month was a motor aura), VAS 10/10, and a MIDAS of 146, including motor and sensory deficits, and had been a non-responder to multiple preventive treatments, including topiramate and amitriptyline. After starting treatment with galcanezumab, there was a substantial and stable reduction in attack frequency and intensity, with only two episodes of visual aura per month (VAS 5–6/10), and no further motor auras. Moreover, there was a dramatic reduction of MIDAS score to 4, and the attacks with motor aura disappeared over one year. Notably, no rebound was observed after treatment discontinuation after one month [16].

Danno et al. presented a case series of six patients with HM, four sporadic and two familial, all treated with galcanezumab. After three months of treatment, reductions in MHDs of at least moderate severity were observed in three patients, while motor weakness improved in four patients and completely resolved in two. MIDAS improved in five of the six patients, and PGIC scores also showed a clinical benefit. However, it is interesting to point out that two patients showed increases in motor aura frequency, and one case presented a *de novo* visual aura after galcanezumab that disappeared after switching to fremanezumab [17].

Another study by Sottani et al. reported a case series of three family members with HM with PRRT2 mutations treated with galcanezumab. After six months of therapy, all three demonstrated substantial clinical improvement, and aura frequency was reduced by at least 50%, with shortened post-ictal phases and improved responsiveness to acute medications. The MIDAS score improved from severe disability to minimal. No adverse events were reported [18].

Héja and Oláh reported a case of a 53-year-old woman with sporadic HM, having a phenotype of chronic migraine and medication overuse headache successfully treated with fremanezumab. In this case, the patient initially underwent unnecessary thrombolysis on two occasions. She had a disabling aura with aphasia, sensory, and motor deficits. Initially, valproic acid was prescribed and then suspended for skin rash, followed by topiramate suspended for dizziness, and finally, fremanezumab was started. After 12 weeks, MHDs fell from ~ 17/month to 3–4/month, attacks were shorter and milder, non-steroidal anti-inflammatory drugs (NSAIDs) use dropped from > 14 to ~ 2 days/month, MIDAS score improved from 29 to 5, and notably, no hemiplegic aura recurred during 11 months of follow-up [19].

Finally, Indelicato et al. reported a large, monocentric cohort of 41 patients with non-polyglutamine *CACNA1A* variants, followed prospectively for up to 20 years. While most patients required interval prophylaxis with standard of care and off-label drugs (including acetazolamide, flunarizine, 4-aminopyridine, and topiramate), only one elderly patient with a treatment-resistant form was treated with galcanezumab, achieving a reduction of MHDs from 4/month to 1/month with good tolerability [20].

While therapeutic benefit was observed across both forms, no subgroup-specific conclusions can be drawn due to the small sample size, the heterogeneous genetic background of familial cases, and the non-systematic reporting of outcomes across studies.

### Risk of bias

All included case reports were judged to have a critical risk of bias, mainly due to incomplete reporting and lack of detail in outcome assessment. Case series were generally rated as having a serious risk of bias. The prospective cohort study was judged to have a serious risk of bias as well. The GRADE table is reported in the Supplementary material.

### Description of the cases

Given that most of the included studies were case reports or case series, the initial certainty of evidence was considered low and was further downgraded to very low due to serious or critical risk of bias, indirectness, and imprecision. As recommended by GRADE, the overall certainty of the body of evidence for each outcome was therefore rated as very low, meaning that the true effect is likely to be substantially different from the reported findings.

### Quantitative synthesis

**Fig. 2:**
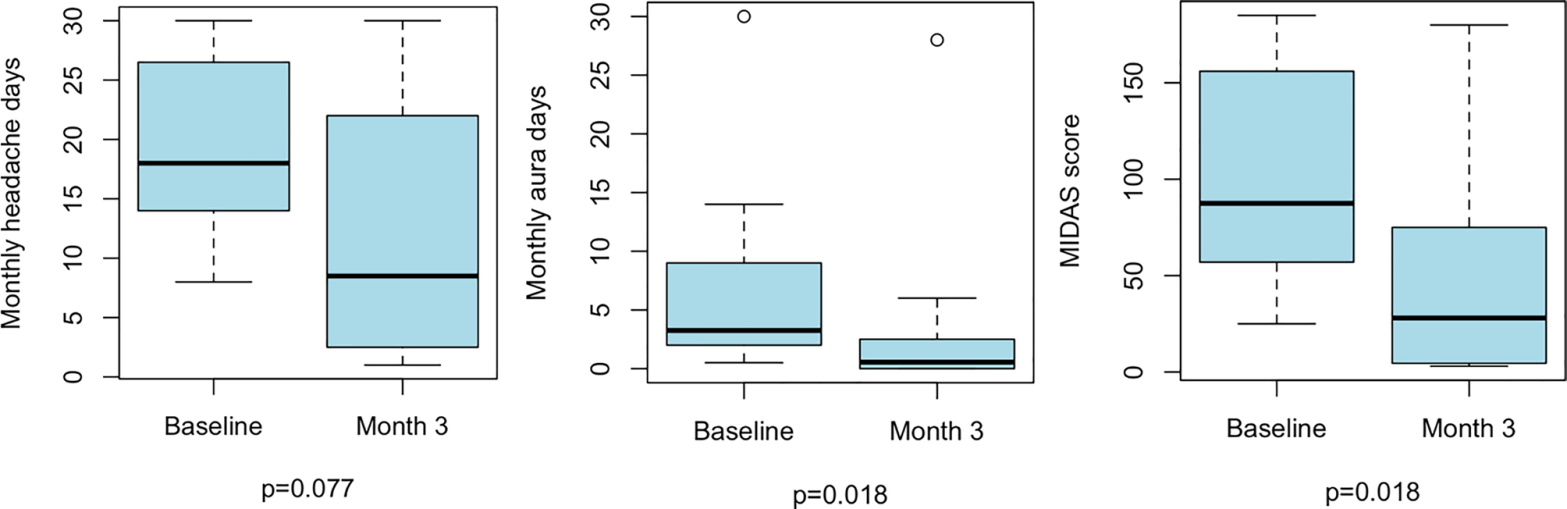
Boxplots showing changes from baseline to month 3 in monthly headache days, monthly aura days, and MIDAS score. The central line represents the median, the box limits indicate the interquartile range (IQR), and whiskers extend to 1.5× IQR

Twelve patients out of 13 were included in the quantitative analysis. The analysis of 12 patients across six studies showed that the median number of MHDs decreased from 18 (IQR 15–25) at baseline to 9 (IQR 3–20) at three months; however, the numerical decrease was not statistically significant (*p* = 0.077). The median number of days with aura decreased from 3 (IQR 2–8) to 0.6 (IQR 0-2.3; *p* = 0.018), and the median MIDAS score decreased from 88 (IQR 66–154) to 28 (IQR 5–74; *p* = 0.018; Fig. 2). Ten out of 12 patients (83.3%, 95% CI 40.0-100.0) reported a > 50% reduction in MHDs and in days with aura, while 9 (75.0%, 95% CI 34.3–100.0) reported a > 50% reduction in the MIDAS score (Fig. 2).

## Discussion

In this systematic review and quantitative single-patient analysis, we evaluated the available evidence on the effectiveness and safety of anti-CGRP mAbs in patients with HM (both sporadic and familial). The identified studies were case reports or small case series, and the certainty of evidence was rated as very low according to the GRADE criteria. Despite these limitations, the results consistently suggested a potential benefit of anti-CGRP mAbs in reducing aura frequency and disability, with a favourable safety profile and a trend toward reduced headache days, albeit not statistically significant.

A timely initiation of preventive treatment during the disease course may be crucial in patients experiencing severe attacks, particularly when accompanied by prolonged neurological deficits [5].

The treatment strategies for HM are empirical, considering that patients with HM have been excluded from clinical trials. Drugs effective in migraine with or without aura are used in HM, including flunarizine and lamotrigine, but other drugs that are usually ineffective in migraine without aura could be useful in HM, such as acetazolamide or verapamil [4]. However, the results of these treatments are inconsistent, and several patients report adverse events and/or the ineffectiveness of multiple treatment lines [2].

In this context, there is a strong need for novel preventive (and acute) therapies that are both effective and safe in this population. Anti-CGRP drugs are increasingly used in patients with migraine with typical aura, with data coming from real-world studies and post-hoc analyses of RCTs [9, 21, 22], with a similar effectiveness in patients with and without aura, reducing in parallel the number of migraine attacks with and without aura [11, 23, 24]. Furthermore, real-world data demonstrated a reduced incidence, intensity, and duration of the aura phenomenon regardless of the anti-CGRP mAb used [11, 23, 24], with some studies also showing a reduction in the number of auras or complete freedom from aura regardless of responder status [10, 25, 26].

Nevertheless, important questions are unanswered regarding the role of CGRP in aura, how CGRP antagonism may influence aura pathophysiology, and to what extent these drugs can affect a central symptom, which is mechanistically linked to CSD, with the knowledge that anti-CGRP mAbs poorly cross the intact blood-brain barrier (BBB) due to their dimension and pharmacokinetic features [8, 27–29].

Emerging evidence supports an involvement of CGRP in aura, although the underlying mechanisms have not yet been fully elucidated [8]. Preclinical and clinical evidence suggest that CSD might activate both peripheral and central components of the trigeminovascular system [30, 31]. CSD has been found to release CGRP from trigeminal fibers in the dura, where it triggers neurogenic inflammation, or within cerebral blood vessels, thus affecting vascular tone [30]. Aligning with these findings, endogenous CGRP was released in a calcium-dependent manner in rat cortical brain slices by CSD [32], and sensory denervation in the meningeal tissue eliminated the CSD-dependent neurogenic inflammatory response [32]. Conversely, CGRP may influence CSD, as intracerebral ventricular perfusion of an anti-CGRP antibody in mice reduced susceptibility to CSD [33].

Other data comes from functional neuroimaging studies (fMRI), demonstrated that both galcanezumab and erenumab are associated with measurable (direct or indirect) central effects, with a reduction of hypothalamic and trigeminal nociceptive network activation. Considering the negligible BBB permeability of CGRP monoclonal antibodies, the effects could be indirectly driven [34].

Lastly, even to a lesser extent, CGRP provocation experiments in humans have also been done in patients with aura [35]. In patients who experienced only migraine attacks with typical aura, CGRP infusion triggered migraine-like attacks without aura in 57% of patients, with four (28%) reporting typical aura symptoms [36, 37].

In this context, HM can be considered a “human model” of aura, which, although atypical, provides a valuable framework for studying the effects of this drug on aura phenomena. Moreover, it offers a potential therapeutic option for a rare and often neglected group of patients who may experience severe complications related to prolonged ictal events. Given the limited availability of data, it is challenging to determine whether treatment response is associated with a specific mutation or with the type of anti-CGRP mAb (subcutaneous versus intravenous) or the pharmacodynamic (ligand vs. receptor), considering that no patients received erenumab, an anti-CGRP receptor.

Concerns have been raised about the possible effects of anti-CGRP mAbs on cerebral hemodynamics, given the established vasodilatory role of CGRP. Available clinical evidence, however, indicates that these antibodies do not exert direct effects on intracranial circulation [38]. Furthermore, evidence from RCTs and real-world data does not report any significant cardiovascular events. To note, no adverse events of any type have been reported in the patients included [39, 40].

The main strength of our work lies in being the first systematic study to synthesize the evidence on the use of anti-CGRP mAbs in HM, a rare and disabling condition with limited therapeutic options and an elevated burden of disease. The study adhered to PRISMA guidelines and used the GRADE approach to evaluate the quality of evidence.

The major limitation of this study is the nature of the included evidence, which consisted predominantly of case reports and small case series with serious-to-critical risk of bias, heterogeneous outcome reporting, and a lack of randomization, control groups, and placebo comparisons. These methodological limitations may have contributed to the inability to detect a statistically significant difference in MHDs, despite a numerical reduction after treatment with anti-CGRP mAbs.

Publication bias cannot be excluded, as positive cases are more likely to be reported. The overall quality of evidence was low, which substantially limits the strength of the conclusions.

In HM, not only headache but also aura symptoms impair daily functioning. However, objective and standardized tools for quantifying aura frequency, severity, and duration in HM are currently lacking, underscoring the need for the development and implementation of uniform assessment metrics in future studies.

There is a need to establish prospective registries and multicentric cohort studies of the treatment of patients with HM, including anti-CGRP drugs, to provide more robust data on effectiveness, safety, and long-term outcomes.

## Supplementary Information

Below is the link to the electronic supplementary material.

Supplementary Material 1

## Data Availability

No datasets were generated or analysed during the current study.

## References

[1] Headache Classification Committee of the International Headache Society (IHS) (2018) The International Classification of Headache Disorders, 3rd edition. Cephalalgia. 38(1):1–211 10.1177/0333102417738202 29368949

[2] Russell MB, Ducros A (2011) Sporadic and Familial hemiplegic migraine: pathophysiological mechanisms, clinical characteristics, diagnosis, and management. Lancet Neurol 10(5):457–470 21458376 10.1016/S1474-4422(11)70048-5

[3] Riant F, Roze E, Barbance C, Méneret A, Guyant-Maréchal L, Lucas C et al (2012) PRRT2 mutations cause hemiplegic migraine. Neurology 79(21):2122–2124 23077016 10.1212/WNL.0b013e3182752cb8

[4] Di Stefano V, Rispoli MG, Pellegrino N, Graziosi A, Rotondo E, Napoli C et al (2020) Diagnostic and therapeutic aspects of hemiplegic migraine. J Neurol Neurosurg Psychiatry 91(7):764–771 32430436 10.1136/jnnp-2020-322850 PMC7361005

[5] Romozzi M, Primiano G, Rollo E, Travaglini L, Calabresi P, Servidei S et al (2021) CACNA1A-p.Thr501Met mutation associated with Familial hemiplegic migraine: a family report. J Headache Pain 22(1):85 34320921 10.1186/s10194-021-01297-5 PMC8317284

[6] Romozzi M, Spartano S, L’Erario FF, Iannone LF, Trigila V, Gentile A et al (2025) Clinical characterization of a novel ATP1A2 p.Gly615Glu mutation in nine family members with Familial hemiplegic migraine. Brain Commun 7(1):fcae447 39723107 10.1093/braincomms/fcae447 PMC11668175

[7] Danno D, Shanahan P, Matharu M (2025) Clinical characteristics of hemiplegic migraine: a clinical study of 163 cases in a tertiary care headache centre. Rinsho Shinkeigaku 65(5):338–351 40301022 10.5692/clinicalneurol.cn-002070

[8] Romozzi M, Calabresi P (2025) Is there a role of calcitonin gene-related peptide in cortical spreading depression mechanisms?– argument pro. J Headache Pain 26(1):90 40295905 10.1186/s10194-025-02011-5 PMC12036227

[9] Ashina M, McAllister P, Cady R, Hirman J, Ettrup A (2022) Efficacy and safety of eptinezumab in patients with migraine and self-reported aura: post hoc analysis of PROMISE-1 and PROMISE-2. Cephalalgia 42(8):696–704 35302389 10.1177/03331024221077646 PMC9218409

[10] Ashina S, Melo-Carrillo A, Toluwanimi A, Bolo N, Szabo E, Borsook D et al (2023) Galcanezumab effects on incidence of headache after occurrence of triggers, premonitory symptoms, and aura in responders, non-responders, super-responders, and super non-responders. J Headache Pain 24(1):26 36927366 10.1186/s10194-023-01560-x PMC10018924

[11] Iannone LF, De Cesaris F, Ferrari A, Benemei S, Fattori D, Chiarugi A (2022) Effectiveness of anti-CGRP monoclonal antibodies on central symptoms of migraine. Cephalalgia 42(13):1323–1330 35775208 10.1177/03331024221111526

[12] Romozzi M, Burgalassi A, Vollono C, Albanese M, Vigani G, De Cesaris F et al (2024) Prospective evaluation of aura during anti-calcitonin gene-related peptide monoclonal antibody therapy after 52 weeks of treatment. Confinia Cephalalgica. 34(1)

[13] Page MJ, McKenzie JE, Bossuyt PM, Boutron I, Hoffmann TC, Mulrow CD et al (2021) The PRISMA 2020 statement: an updated guideline for reporting systematic reviews. BMJ 372:n71 33782057 10.1136/bmj.n71 PMC8005924

[14] Ouzzani M, Hammady H, Fedorowicz Z, Elmagarmid A (2016) Rayyan—a web and mobile app for systematic reviews. Syst Reviews 5(1):210 10.1186/s13643-016-0384-4 PMC5139140 27919275

[15] Antenucci P, Pes F, Cesnik E, Capone JG, Padroni M (2025) A case of sporadic hemiplegic migraine treated with eptinezumab: should we consider anti-CGRP antibodies for selected patients? Neurol Sci 46(6):2883–2885 39745588 10.1007/s10072-024-07980-0

[16] D’Apolito M, Rispoli MG, Ajdinaj P, Travaglini D, Bonanni L (2024) Sporadic hemiplegic migraine with novel missense mutation in the SCN1A gene and positive response to anti-CGRP antibody: a case report. Neurol Sci 45(11):5535–5537 38940877 10.1007/s10072-024-07665-8

[17] Danno D, Ishizaki K, Kikui S, Takeshima T (2023) Treatment of hemiplegic migraine with anti-calcitonin gene-related peptide monoclonal antibodies: a case series in a tertiary-care headache center. Headache 10.1111/head.14591 37366160

[18] Sottani C, Di Lazzaro G, Calabresi P, Pomponi MG, Tiziano FD, Bentivoglio AR et al (2025) Efficacy of galcanezumab in proline-rich transmembrane protein 2 (PRRT2)-associated Familial hemiplegic migraine: A case series. Headache 65(2):377–381 39345003 10.1111/head.14840 PMC11794975

[19] Héja M, Oláh L (2025) Efficacy of anti-calcitonin gene-related peptide monoclonal antibodies in hemiplegic migraine: a case report and review of literature. Front Neurol 16:1579203 40264646 10.3389/fneur.2025.1579203 PMC12011815

[20] Indelicato E, Nachbauer W, Amprosi MS, Maier S, Unterberger I, Delazer M et al (2024) Natural history of non-polyglutamine CACNA1A disease in Austria. J Neurol 271(10):6618–6627 39110218 10.1007/s00415-024-12602-y PMC11446988

[21] Igarashi H, Shibata M, Ozeki A, Matsumura T (2023) Galcanezumab effects on migraine severity and symptoms in Japanese patients with episodic migraine: secondary analysis of a phase 2 randomized trial. Neurol Ther 12(1):73–87 36266558 10.1007/s40120-022-00410-3 PMC9837349

[22] Ashina M, Goadsby PJ, Dodick DW, Tepper SJ, Xue F, Zhang F et al (2022) Assessment of erenumab safety and efficacy in patients with migraine with and without aura: A secondary analysis of randomized clinical trials. JAMA Neurol 79(2):159–168 34928306 10.1001/jamaneurol.2021.4678 PMC8689443

[23] Straube A, Stude P, Gaul C, Schuh K, Koch M (2021) Real-world evidence data on the monoclonal antibody erenumab in migraine prevention: perspectives of treating physicians in Germany. J Headache Pain 22(1):133 34742252 10.1186/s10194-021-01344-1 PMC8572451

[24] Mahović D, Bračić M, Jakuš L, Vukovic Cvetkovic V, Krpan M (2022) Effectiveness and safety of erenumab in chronic migraine: A Croatian real-world experience. Clin Neurol Neurosurg 214:107169 35151970 10.1016/j.clineuro.2022.107169

[25] Matteo E, Pensato U, Favoni V, Giannini G, Pierangeli G, Cevoli S (2021) Do anti-CGRP drugs have a role in migraine aura therapy? J Neurol 268(6):2273–2274 33856547 10.1007/s00415-021-10546-1

[26] Albanese M, Mercuri NB (2022) Could the new anti-CGRP monoclonal antibodies be effective in migraine aura? Case reports and literature review. J Clin Med, 11(5) 10.3390/jcm11051228 PMC8911201 35268319

[27] Noseda R, Schain AJ, Melo-Carrillo A, Tien J, Stratton J, Mai F et al (2020) Fluorescently-labeled fremanezumab is distributed to sensory and autonomic ganglia and the dura but not to the brain of rats with uncompromised blood brain barrier. Cephalalgia 40(3):229–240 31856583 10.1177/0333102419896760 PMC7233263

[28] Edvinsson L, Haanes KA, Warfvinge K, Krause DN (2018) CGRP as the target of new migraine therapies — successful translation from bench to clinic. Nat Reviews Neurol 14(6):338–350 10.1038/s41582-018-0003-1 29691490

[29] Johnson KW, Morin SM, Wroblewski VJ, Johnson MP (2019) Peripheral and central nervous system distribution of the CGRP neutralizing antibody [(125)I] galcanezumab in male rats. Cephalalgia 39(10):1241–1248 31003588 10.1177/0333102419844711

[30] Close LN, Eftekhari S, Wang M, Charles AC, Russo AF (2019) Cortical spreading depression as a site of origin for migraine: role of CGRP. Cephalalgia 39(3):428–434 29695168 10.1177/0333102418774299 PMC7007998

[31] Russo AF, Hay DL (2023) CGRP physiology, pharmacology, and therapeutic targets: migraine and beyond. Physiol Rev 103(2):1565–1644 36454715 10.1152/physrev.00059.2021 PMC9988538

[32] Tozzi A, de Iure A, Di Filippo M, Costa C, Caproni S, Pisani A et al (2012) Critical role of calcitonin gene-related peptide receptors in cortical spreading depression. Proc Natl Acad Sci U S A 109(46):18985–18990 23112192 10.1073/pnas.1215435109 PMC3503217

[33] Eftekhari S, Kechechyan GM, Faas G, Charles A (eds) (2017) The CGRP receptor antagonist olcegepant modulates cortical spreading depression in vivo. Cephalalgia

[34] Basedau H, Sturm LM, Mehnert J, Peng KP, Schellong M, May A (2022) Migraine monoclonal antibodies against CGRP change brain activity depending on ligand or receptor target - an fMRI study. eLife. 11 10.7554/eLife.77146 PMC9126581 35604755

[35] Ashina H, Schytz HW, Ashina M (2018) CGRP in human models of primary headaches. Cephalalgia 38(2):353–360 27940880 10.1177/0333102416684344

[36] Hansen JM, Hauge AW, Olesen J, Ashina M (2010) Calcitonin gene-related peptide triggers migraine-like attacks in patients with migraine with aura. Cephalalgia 30(10):1179–1186 20855363 10.1177/0333102410368444

[37] Al-Khazali HM, Ashina H, Wiggers A, Rose K, Iljazi A, Christensen RH et al (2023) Calcitonin gene-related peptide causes migraine aura. J Headache Pain 24(1):124 37679723 10.1186/s10194-023-01656-4 PMC10483878

[38] Altamura C, Viticchi G, Fallacara A, Costa CM, Brunelli N, Fiori C et al (2021) Erenumab does not alter cerebral hemodynamics and endothelial function in migraine without aura. Cephalalgia 41(1):90–98 32867533 10.1177/0333102420956692

[39] Messina R, Huessler E-M, Puledda F, Haghdoost F, Lebedeva ER, Diener H-C (2023) Safety and tolerability of monoclonal antibodies targeting the CGRP pathway and gepants in migraine prevention: A systematic review and network meta-analysis. Cephalalgia 43(3):03331024231152169 10.1177/03331024231152169 36786548

[40] Sun W, Li Y, Xia B, Chen J, Liu Y, Pang J et al (2023) Adverse event reporting of four anti-Calcitonin gene-related peptide monoclonal antibodies for migraine prevention: a real-world study based on the FDA adverse event reporting system. Front Pharmacol 14:1257282 38264523 10.3389/fphar.2023.1257282 PMC10803415

