# Supplementary material for: Effectiveness and safety of anti-CGRP monoclonal antibodies in hemiplegic migraine: an individual patient quantitative analysis
Source: J Headache Pain. 2026 Jan 30;27(1):56. doi: 10.1186/s10194-026-02283-5 (PMC12930611; doi:10.1186/s10194-026-02283-5)
Supplement: Supplementary file 1 — Supplementary Material 1 [file 10194_2026_2283_MOESM1_ESM.docx]

| **Study** | **Study Design** | **Risk of Bias** | **Inconsistency** | **Indirectness** | **Imprecision** | **Other Considerations** | **Certainty of Evidence** |
| --- | --- | --- | --- | --- | --- | --- | --- |
| Antenucci et al. 2025 | Case report | Critical | Serious | Not serious | Critical | None | Very low |
| D'Apolito et al. 2024 | Case report | Critical | Serious | Not serious | Critical | None | Very low |
| Danno et al. 2022 | Case series | Serious | Serious | Not serious | Critical | None | Very low |
| Sottani et al. 2024 | Prospective case series | Serious | Not serious | Not serious | Serious | None | Very low |
| Heja and Olah 2025 | Case report | Critical | Serious | Not serious | Critical | None | Very low |
| Indelicato et al. 2024 | Prospective cohort | Serious | Not serious | Not serious | Serious | None | Very low |
